# Altered IgG4 serum levels in VEXAS syndrome – a retrospective monocentric cohort study

**DOI:** 10.1007/s00296-025-05824-4

**Published:** 2025-03-22

**Authors:** Sebastian J. Saur, Benedikt Obermaier, Reinhild Klein, Matthias Hahn, Falko Fend, Sven Mattern, Joerg C. Henes, Ann-Christin Pecher

**Affiliations:** 1 Department of Internal Medicine II (Haematology, Oncology, Immunology and Rheumatology), University Hospital of Tuebingen, Otfried-Mueller-Strasse 10, 72076 Tuebingen, Germany; 2 Department of Dermatology, University Hospital of Tuebingen, Tuebingen, Germany; 3 Institute of Pathology and Neuropathology and Comprehensive Cancer Center, University Hospital of Tuebingen, Tuebingen, Germany

**Keywords:** VEXAS syndrome, Autoinflammatory diseases, Myelodysplastic syndromes, Immunoglobulin G4-related disease, UBA1 protein, human

## Abstract

VEXAS (vacuoles, E1 enzyme, X-linked, autoinflammatory, somatic) syndrome is an autoinflammatory disorder characterized by somatic mutations in the *UBA1* gene in hematopoietic stem cells and associated with diffuse inflammation and myelodysplastic changes amongst others. Due to unspecific symptoms the diagnosis is challenging, and there is an unmet need for clinical markers to select patients for genetic examination. Sera of 9 patients with confirmed VEXAS syndrome were analyzed for immunoglobulin (Ig)G4 levels. Disease parameters and clinical response to therapy were correlated with IgG4 levels. A histopathological examination was performed on the available samples to exclude IgG4-related autoimmune diseases. In this cohort, 44% of our patients showed markedly elevated serum IgG4 levels. We observed a general trend toward a positive correlation between IgG4 levels and inflammatory markers as well as a correlation with clinical response in one patient. Histopathological analysis showed no evidence of IgG4 related disease. IgG4 levels seem to be elevated in a relevant fraction of patients with VEXAS syndrome. In some cases, this might be misinterpreted as IgG4-related disease, a pitfall clinicians should be aware of. Furthermore, our results warrant the further evaluation of a potential correlation of IgG4 levels with disease activity and severity of inflammation. IgG4 serum levels might be useful in the evaluation of the disease course.

## Introduction

VEXAS (vacuoles, E1 enzyme, X-linked, autoinflammatory, somatic) syndrome is a recently identified autoinflammatory disorder caused by somatic mutations in the *UBA1* gene in hematopoietic stem cells [1]. It has been suggested, that *UBA1* mutations associated with VEXAS syndrome result in catalytically impaired isoforms of the ubiquitin-like modifier activating enzyme 1 (UBA1) and consequently trigger an inflammatory response through the unfolded protein response [1–4]. As the disease presents with a wide range of nonspecific symptoms, including “B symptoms”, skin rash, cytopenia, and arthralgias, diagnosis is challenging and often delayed [5]. In several cases, patients initially diagnosed with giant cell arthritis, relapsing polychondritis, polyarteritis nodosa, sweet syndrome, ANCA-associated vasculitis and myelodysplastic syndrome (MDS) were later rediagnosed with VEXAS syndrome [5–9]. Therefore, there is a clear need to identify diagnostic markers and criteria for genetic diagnostics, especially since *UBA1* mutations seem to be relatively common, with a recently reported frequency of 1 in 4,269 men older than 50 years [10].

Immunoglobulin (Ig)G4 is the least common subclass of serum IgG and has special immunological characteristics, including low affinity for complement and Fc receptors, and the ability to undergo fragment antigen binding (Fab)-arm exchange, which enables bispecific antigen binding [11]. Abnormal serum-IgG4 levels are associated with various diseases: infections, allergies, autoimmune diseases as well as disrupted tumor response. Especially, repeated antigen exposure has been connected to this IgG subtype [11]. Furthermore, IgG4 serum levels are already widely used as a diagnostic marker for IgG4-related diseases [12, 13]. Individual cases of patients initially diagnosed with IgG4-related disease who were later diagnosed with VEXAS syndrome have been described in the literature. Relevantly elevated IgG4 serum levels were detected in these patients [14, 15] and 2 further recently reported cases [16].

Here we report elevated IgG4 serum levels in a series of VEXAS patients and discuss a potential role of IgG4 as a diagnostic indicator for VEXAS syndrome.

## Methods

This monocentric study addresses the serum level of IgG4 in patients with VEXAS syndrome. The reported cohort includes all patients with VEXAS syndrome that were diagnosed and treated in our department of internal medicine or department of dermatology at the University Hospital of Tuebingen, Germany until January 2024. We report 9 patients in whom different pathogenic *UBA1* mutations have been detected and who were diagnosed with VEXAS syndrome. The frequency of *UBA1* mutations was distributed as follows: c.121A > G, p.Met41Val (n = 2); c.122 T > C, p.Met41Thr (n = 4); c.121A > C, p.Met41Leu (n = 2); c.118G > C, p.(splice) (n = 1). All patients were male, median age was 72 years. Patients’ clinical disease parameters and other details are summarized in Table 1. All patients had already started immunosuppressive treatment prior to being referred to our center and before the collection of the first sample for IgG4 diagnostics. The available serum samples had been collected in the time period April 2018 to January 2024.

**Table 1:** Clinical characteristics of patients with VEXAS syndrome

Patient	1	2	3	4	5	6	7	8	9
Age (years)	65	72	74	75	78	72	73	62	65
*UBA1* mutation	c.121A > G p.Met41Val VAF 65%	c.118G > C p.(splice) VAF 62%	c.122 T > C p.Met41Thr	c.121A > C p.Met41Leu VAF 73%	c.122 T > C p.Met41Thr VAF 55%	c.121A > G p.Met41Val VAF 54%	c.122 T > C pMet41Thr VAF 64%	c.121A > C p.Met41Leu VAF 83%	c.122 T > C p.Met41Thr
Macrocytic anaemia	+	+	+	+	+	+	+	+	+
Bone marrow abnormalities	Cytopenia, dysplasia	MGUS, plasma cell proliferation	Plasma cell proliferation	–	Plasma cell proliferation, dysplasia	Dysplasia	Dysplasia, plasma cell proliferation	Hypercellular bone marrow, dysplasia	Hypercellular bone marrow, no dysplasia
Highest serum IgG4 level (mg/dl)	14	22	**216**	**290**	**288**	**219**	38	34	92
Elevation of other IgG subclasses	-	IgG1	IgG2	**-**	IgG1, IgG2	–	–	–	–
Chondritis	Ear	Nose, ear	–	–	Ear	–	Ear	–	-
B symptoms	–	+	+	+	–	+	+	–	+
Arthralgia/ arthritis	+	+	–	+	+	+	+	–	+
Skin manifestation	Rash, erythema nodosum	Pruritus, panniculitis	Rash, panniculitis	Erythema nodosum, leukocyctoclastic vasculitis	Neutrophilic dermatosis	Erythema nodosum	Rosacea	Neutrophilic dermatosis	Erythema nodosum
Eye manifestation	Conjunctivitis	Conjunctivitis	–	Venous vascular occlusion	Recurrent conjunctivitis	Inflammation of retrobulbar fat tissue	–	–	–
Lung manifestation	Disseminated nodules	Ground-glass opacities	Disseminated nodules, fibrotic reticulation	Pleural effusion	Isolated nodules, ground-glass opacities	Diffuse areas of consolidation	Disseminated nodules	–	–
Thrombotic events	Thrombophlebitis, thrombosis	–	Thrombophlebitis, thrombosis	Venous thrombosis of the eye, myocardial infarction	Thrombosis	Thrombophlebitis, ventricular apex thrombus	–	–	Thrombophlebitis, thrombosis

*Ig* immunoglobulin, *MGUS* monoclonal gammopathy, *VAF* variant allele frequency, *UBA1* ubiquitin activating enzyme 1. Elevated levels of IgG4 are indicated in bold

Serum for IgG4 was separated and stored at − 20°C until used for assay. IgG4 was measured using SPAPLUS® system and Optilite Subclass Assays for IgG subclasses from Binding Site (Birmingham, United Kingdom).

Medical record data extraction included ongoing immunosuppressant therapy, inflammation markers (C-reactive protein [CRP]), erythrocyte sedimentation rate (ESR), as well as hemoglobin (Hb), mean corpuscular volume (MCV) and clinical manifestations of VEXAS syndrome.

A histopathological examination was performed on the available tissue samples (bone marrow, pancreas, lung, skin). The morphology, cytology, and immunohistochemical staining for MUM1, IgG, IgG4 were evaluated in formalin-fixed, paraffin-embedded (FFPE) tissue sections. All immunohistochemical stains are established routine protocols which are used in routine diagnostics.

The study was conducted in accordance with the Declaration of Helsinki and was approved by the local ethics committee of the medical faculty of the University of Tuebingen and the University Hospital of Tuebingen (747/2023BO2, 18.12.2023).

## Results

### Serum IgG4 levels in patients with VEXAS

**Fig. 1:**
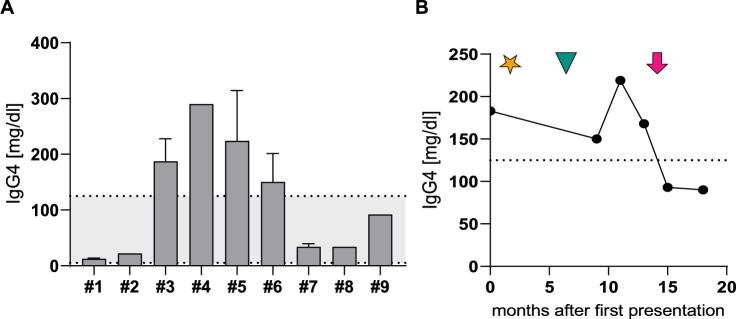
IgG4 levels in patients with VEXAS syndrome characterized by UBA1 mutation. **A** Serum levels of IgG4 in 9 consecutive patients (#1-#9) at random timepoints. For patient #2, #4, #8, #9 only one timepoint was available. The dotted line with grey area highlights normal values for IgG4. **B** IgG4 levels in the course of patient #6 under therapy. The dotted line shows the upper cut off for normal IgG4 levels. Pink arrow highlights timepoint of clinical remission (defined as no B-symptoms, no arthralgias, normalized CRP levels) after initiation of anakinra. Prior unsuccessful therapies are highlighted by yellow star (rituximab) and green arrowhead (mycophenolate mofetil). Dotted line shows upper-limit of normal IgG4 levels

In this cohort of 9 patients who were diagnosed with VEXAS syndrome at our clinic, 4 patients (44%) showed relevantly elevated IgG4 serum levels (Fig. 1A). From patient #6, several sequential samples were available, allowing for a comparison of IgG4 levels in relation to therapy and clinical remission (Fig. 1B). This patient had previously been diagnosed with an IgG4-related disease and had never shown a satisfactory clinical response to prior therapy with persistent high corticosteroid dependence (prednisone equivalent > 15 mg/day). Interestingly, IgG4 levels only dropped after clinical remission (no B-symptoms, no arthralgias, normalized CRP levels) in response to initiation of anakinra and stayed low despite corticosteroid reduction (dosing at last measured timepoint: prednisone equivalent 5 mg/day).

### Correlation with disease parameters

**Fig. 2:**
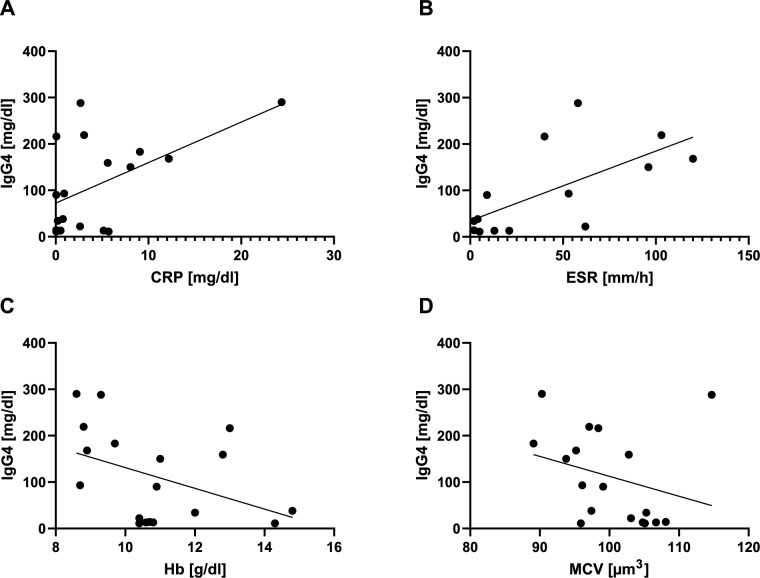
Correlation for IgG4 serum levels with various inflammatory markers and clinical parameters of VEXAS syndrome. The correlation coefficient (R^2^) was **A** 0.28 for C-reactive protein (CRP) **B** 0.41 for erythrocyte sedimentation rate (ESR) **C** 0.16 haemoglobin (Hb) and **D** 0.067 for mean corpuscular volume (MCV)

As we observed a normalization of serum IgG4 levels after clinical remission in the case of patient #6, we further investigated potential correlations to typical disease parameters (Fig. 2). CRP and ESR demonstrated a positive correlation (*R*^2^ = 0.29 and *R*^2^ = 0.43, respectively). Hb levels and MCV showed an inverse relationship and medium to low correlation levels (*R*^2^ = 0.18 and *R*^2^ = 0.08, respectively). However, considering the low number of patients and samples, it is not possible to draw substantial conclusions based on these trends.

**Fig. 3:**
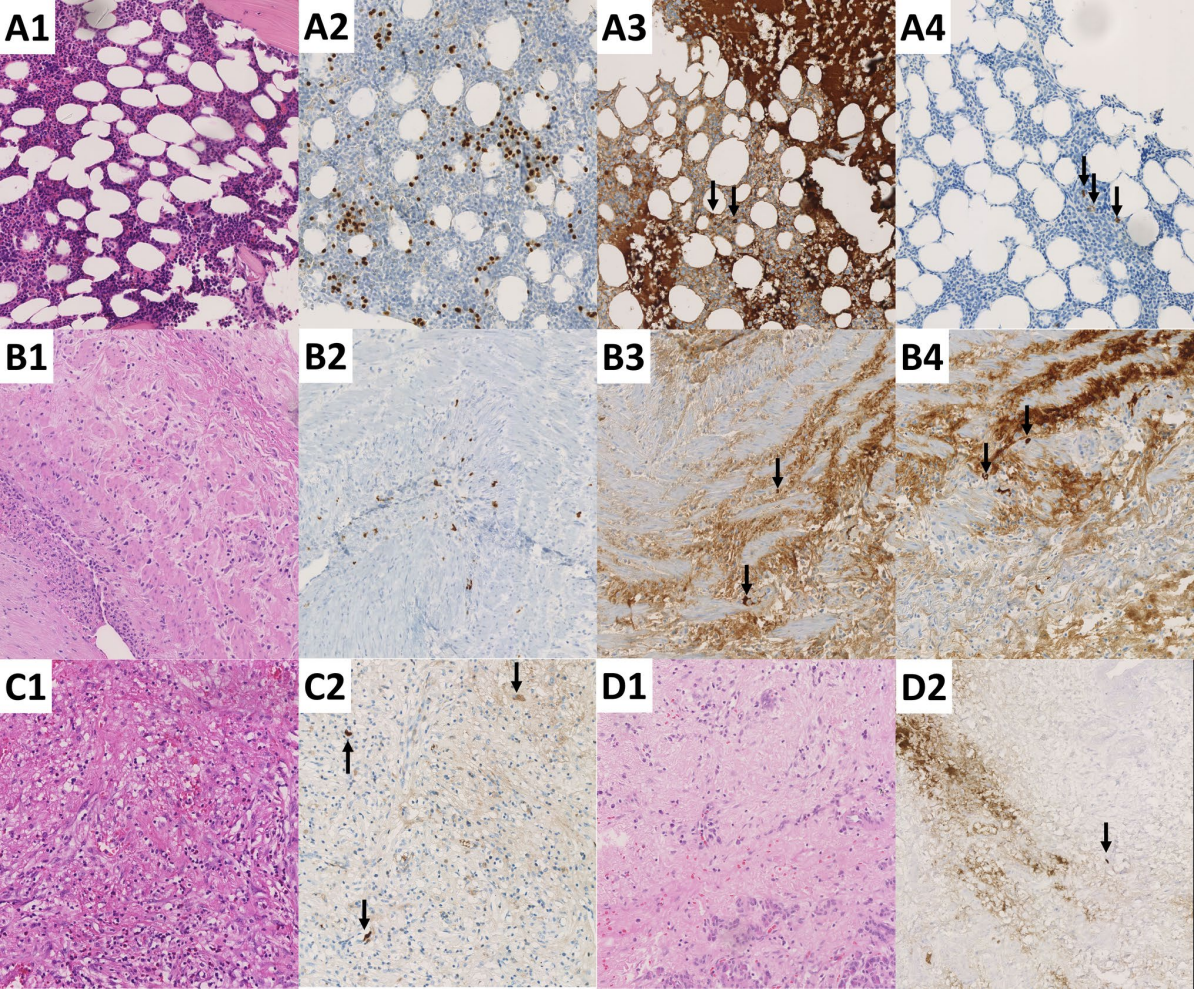
Histological images of different tissue sample throughout the history of patient #3 (2015 to 2023). **A** H&E (A1) staining of a core needle biopsy with hypercellular bone marrow. MUM1 (A2) displaying an increased amount of plasma cells. IgG (A3) only stains a small subset of the plasma cells (→), while IgG4 (A4) stains even fewer cells (→). **B** H&E (B1) of a skin biopsy displaying sings of a vasculitis. MUM1 (B2) only marking interspersed plasma cells with only singular intermixed IgG (B3) positive cells with just single cells showing positivity for IgG4 (B4). **C** H&E (C1) staining of a lung resection specimen with an organizing process e.g. post-pneumonia or post-infarction. Only single IgG4-positive (C2) plasma cells are intermixed (→). **D** H&E (D1) of a pancreas tail resection due to a pseudotumor in a known autoimmune pancreatitis. Only single plasma cells (→) displaying positivity for IgG4 (D2). All images: original magnification 200x.

Histopathological examination could exclude IgG4-related autoimmune diseases in all available tissue samples (bone marrow, pancreas, lung, skin; Fig. 3). In 4 cases, bone marrow samples showed plasma cell proliferation (polytypic reactive plasma cells), but this did not concur with the evidence of elevated serum IgG4 levels: only 2 patients with elevated IgG4 levels had signs of plasma cell proliferation.

Statistical analysis was performed in GraphPad Prism. Correlation coefficients were calculated by simple linear regression.

## Discussion

Here we report on a cohort of 9 patients with VEXAS syndrome, of whom 44% showed markedly increased serum IgG4 levels. Preliminary observations suggest a positive trend between CRP, ESR, and IgG4 levels, but the small sample size precludes definitive conclusions.

To our knowledge IgG4 levels of patients with VEXAS Syndrome have so far been only reported in single cases [14–16]. This study provides a comprehensive data set on our monocentric cohort, including IgG4 serum levels of all patients. While our observation sheds light on the potential diagnostic value of IgG4 in the context of VEXAS syndrome, our study is clearly limited by the small size of our cohort and the few available samples. Further research is imperative to confirm our findings and their relevance against the background that elevated IgG4 levels have also been reported for a variety of other autoinflammatory diseases [17].

Although the typical presentations of IgG4-related disease differ in relevant aspects from VEXAS Syndrome, such as a less acute presentation without fever, there is certain overlap of characteristic features in their autoinflammatory manifestations such as skin lesions, constitutional and musculoskeletal symptoms and multi-organ involvement [12, 18–21]. While IgG4-related diseases often respond to therapy with rituximab, this so far has been of limited use in VEXAS syndrome [11]. Based on these observations, we recommend evaluating the possibility of an underlying VEXAS syndrome in patients with suspected IgG4-related disease, particularly in those with atypical clinical courses or failure to rituximab. Such an approach aligns with the evolving landscape of personalized and precision medicine, where identifying specific biomarkers holds the key to tailored diagnostic and therapeutic interventions.

The exact mechanisms by which UBA1 mutations lead to inflammation and pathogenesis remain unknown to date. Ubiquitination is an important step to target proteins for degradation in the proteasome. In VEXAS syndrome UBA1, which catalyzes a crucial step in the ubiquitination process, is functionally impaired which by accumulation of proteins might lead to activation of unfolded protein response and inflammatory response [4, 22].

Lymphatic cells are generally reduced in VEXAS syndrome and changes in T cell compartment of patients VEXAS syndrome, including reduced TCR diversity, have been suggested to be associated with common antigens in VEXAS syndrome [4]. Abnormal protein degradation pathways might result in the expression of novel or aberrant antigens, leading to the presentation of immunogenic epitopes [4]. This could contribute to the activation of B cells resulting in increased IgG4 production. In addition, one could speculate that elevated IgG4 levels in VEXAS patients may also result from dysregulated T-cell responses. The impaired UBA1 function in VEXAS may, in consequence of protein overexpression as well as aberrant antigen expression, also lead to abnormal T-helper 2 (Th2) cell activation, which promotes IgG4 production through cytokines like IL-4 and IL-13 [23, 24].

The dysregulation of ubiquitination may also result in elevated IgG4 levels as part of a broader inflammatory response. IgG4 is known to act as a “regulatory antibody” in chronic inflammation and allergic reactions. Persistent low-grade inflammation in VEXAS might sustain elevated IgG4 levels, as chronic inflammatory conditions often correlate with increased IgG4 production [17].

Elucidating whether the elevated IgG4 levels in VEXAS patients signify a genuine involvement in the pathogenesis or are a consequence of the complex immune dysregulation characteristic of VEXAS is part of future investigations. Thorough investigation of the underlying mechanisms of this observed relationship is required.

In conclusion, the exploration of IgG4 serum levels as a potential diagnostic marker for VEXAS syndrome not only expands our understanding of this still not well understood disorder but also highlights the interconnectedness of seemingly disparate autoimmune conditions. Recognizing the potential mimicry between IgG4-related diseases and VEXAS syndrome is a crucial step toward refining diagnostic strategies and paving the way for more targeted and effective therapeutic interventions in the realm of autoinflammatory disorders.

## Data Availability

The data that support the findings of this study are available on request from the corresponding author (BO). The data are not publicly available due to restrictions, such as containing information that could compromise the privacy of research participants.
